# Supplementary material for: Association of food environment with diet quality and Body Mass Index (BMI) of school-going adolescents in Nepal
Source: PLoS One. 2025 Apr 21;20(4):e0321524. doi: 10.1371/journal.pone.0321524 (PMC12011221; doi:10.1371/journal.pone.0321524)
Supplement: S1 Table — (WORD) [file pone.0321524.s001.docx]

**Table 1: Food groups and the corresponding items**

| **S.N.** | **Food Groups assessed** | | | **Food items included in the group** |
| --- | --- | --- | --- | --- |
| 1. | Starchy staples | Rice, paratha, naan, pau roti, or momo, Roti, whole grain bread, maize, millet, barley, sorghum, buckwheat, or dhindo, Potato, yam, wild yam, or white sweet potato | | |
| 2. | Legumes, Nuts & Seeds | | Daal, chickpeas, beans, soybeans, or quanti, Almonds, peanuts, cashews, pistachios, or walnuts | |
| 3. | Animal Source foods | Eggs, Paneer or cheese,  Dahi, buttermilk, or lassi, Goat, mountain goat, lamb or sheep, buffalo, or yak, Local pig or hybrid pig, Chicken, duck, or pigeon, Fish or dried fish, Milk, tea with milk, or powdered milk | | |
| 4. | Salty/Fried Snacks | Chips, Kurekure, Chisbal, Dalmoth, or Bhujiya, Noodles, Samosa, pakora, sel roti, puri, or tareko khaja | | |
| 5. | Processed Meat | Sausages, ham, bacon, or canned meat | | |
| 6. | Fruits and Vegetables | Carrots or ripe yellow pumpkin, Saag, spinach, mustard greens, fennel greens, pumpkin shoots, taro leaves, or amaranth greens, Gundruk, chamsur palungo, fenugreek greens, or broccoli, Tomatoes, cauliflower, cabbage, gourd, or eggplant, Bitter gourd, bottle gourd, green pumpkin, lady finger, or radish, Papaya, ripe mango, apricots, or persimmon, Orange, pomelo, grapefruit, or kumquat, Apple, banana, avocado, watermelon, mulberries, amla, or guava, Grapes, raisins, peaches, plums, pomegranate, Asian pear, or jackfruit | | |
| 7. | Fiber containing food items | Carrots or ripe yellow pumpkin, Saag, spinach, mustard greens, fennel greens, pumpkin shoots, taro leaves, or amaranth greens, Gundruk, chamsur palungo, fenugreek greens, or broccoli, Tomatoes, cauliflower, cabbage, gourd, or eggplant, Bitter gourd, bottle gourd, green pumpkin, lady finger, or radish, Papaya, ripe mango, apricots, or persimmon, Orange, pomelo, grapefruit, or kumquat, Apple, banana, avocado, watermelon, mulberries, amla, or guava, Grapes, raisins, peaches, plums, pomegranate, Asian pear, or jackfruit, Daal, chickpeas, beans, soybeans, or quanti, Almonds, peanuts, cashews, pistachios, or walnuts, Roti, whole grain bread, maize, millet, barley, sorghum, buckwheat, or dhindo | | |
| 8. | Sugar Sweetened food items | Cakes, biscuits, cookies, donuts, haluwa, jeri/jalebi, Mithai, kheer, chocolates, candies, toffees/ ice cream, Chiya with sugar, coffee with sugar, milk with sugar, Horlicks/Bournvita, Fruit juice, fruit drinks such as Real/ Frooti/ sugar cane juice, Sweet bottled drinks such as Coke, Fanta, Sprite, /energy drinks such as Red Bull | | |
| 9. | Saturated fat containing food items | Mithai, kheer, chocolates, candies, toffees, or ice cream, Paneer or cheese, Dahi, butter milk, or lassi, Sausages, ham, bacon, or canned meat, Goat, mountain goat, lamb or sheep, buffalo, or yak, Local pig or hybrid pig, Chicken, duck, or pigeon, Fish or dried fish, Milk, tea with milk, or powdered milk, KFC, Pizza Hut, or other places that serve pizza or burger | | |
