# Supplementary material for: Association of food environment with diet quality and Body Mass Index (BMI) of school-going adolescents in Nepal
Source: PLoS One. 2025 Apr 21;20(4):e0321524. doi: 10.1371/journal.pone.0321524 (PMC12011221; doi:10.1371/journal.pone.0321524)
Supplement: S2 Annex — (WORD) [file pone.0321524.s002.docx]

**CONSENT FORM**

**Title of the study:** Association of Food Environment with Diet Quality and Body Mass Index (BMI) of the school going adolescents in Budanilkantha Municipality

**Why is this study being done?**

You are being invited to participate in this research study. Kathmandu University School of Medical Sciences is conducting this study in Budanilkantha municipality. This study is aimed at understanding how food affects the dietary quality and Body Mass Index (BMI) of school going adolescents of age 15-19 years. The purpose of this consent form is to help you decide if you want to be in the research study.

**Why am I selected?**

You are being asked to take part in this research study as you fall in the age group (15-19 years) which is our study population chosen after a thorough literature review.

**What will happen if I take part in this research study?**

If you volunteer to participate in this study, you will be asked to sign and date this consent form. The research assistant will provide you with survey questionnaires which will take about thirty minutes to fill up.

**Are there any potential risks or benefits that I can expect from this study?**

There are no known or expected risks or benefits associated with this study.

**What other choices do I have if I choose not to participate?**

You will decide whether you want to participate in this study or not. You can choose whether or not you want to be in this study, and you may withdraw your consent and discontinue participation at any time. Also, you can deny to answer any questions during the interview. Whatever decision you make, there will be no penalty to you, and it will have no effect on any treatment or services you are receiving.

**Will information about me and my participation be kept confidential?**

Study records that identify you will be kept confidential throughout and after the study. Except when required by law, you will not be identified by name, address, telephone number, or any other direct personal identifier in study records outside of KUSMS.

**Costs of participation:**

There are no costs to you for participating in this study.

**Who can I contact if I have questions about this study?**

If you have any questions, queries, suggestions regarding the study please feel free to contact

**Pragya Sharma**

Email:pragyasharma@student.kusms.edu.np

Phone: 9843758178

**Dr. Archana Shrestha**

Email:archana@kusms.edu.np

Phone: 9801002245

For questions about your rights as a research participant, or to discuss problems, concerns or suggestions related to the research, or to obtain information or offer input about the research, please contact Institutional Review Committee (IRC), KUSMS.

**Statement of consent**

"I have read this form and agree for my child's participant in this study."

**Signature of the Participant:**

Date:

Time:

Contact Number of the Participant:

Signature of Person Obtaining Consent:
