# Supplementary material for: Association of food environment with diet quality and Body Mass Index (BMI) of school-going adolescents in Nepal
Source: PLoS One. 2025 Apr 21;20(4):e0321524. doi: 10.1371/journal.pone.0321524 (PMC12011221; doi:10.1371/journal.pone.0321524)
Supplement: S3 Annex — (WORD) [file pone.0321524.s003.docx]

**ASSENT FORM**

**Title of the study:** Association of Food Environment with Diet Quality and Body Mass Index (BMI) of the school going adolescents in Budanilkantha Municipality

**Why is this study being done?**

Your child is being invited to participate in this research study. Kathmandu University School of Medical Sciences (KUSMS) is conducting this study in Budanilkantha municipality. This study is aimed at understanding how food affects the dietary quality and Body Mass Index (BMI) of school going adolescents of age 15-19 years. The purpose of this assent form is to help you decide if you want your child to be in the research study.

**Why is your child selected for this study?**

Your child is being asked to take part in this research study as you fall in the age group (15-19 years) which is our study population chosen after a thorough literature review.

**What will happen if your child takes part in this research study?**

If you volunteer to participate in this study, you will be asked to sign and date this assent form. The research assistant will provide your child with survey questionnaires which will take about thirty minutes to fill up.

**Are there any potential risks or benefits that you and/or your child can expect from this study?**

There are no known or expected risks or benefits associated with this study.

**What other choices do you have if your child chooses not to participate?**

You will decide whether your child wants to participate in this study or not. You can choose whether or not your child to participate in this study, and you may withdraw your consent and discontinue his/her participation at any time. Also, your child can deny to answer any questions during the interview. Whatever decision you make, there will be no penalty to you or your child, and it will have no effect on any treatment or services you or your child are receiving.

**Will information about my child and his/her participation be kept confidential?**

Study records that identify your child will be kept confidential throughout and after the study. Except when required by law, your child will not be identified by name, address, telephone number, or any other direct personal identifier in study records outside of KUSMS.

**Costs of participation:**

There are no costs to you and/or your child for participating in this study.

**Who can I contact if I have questions about this study?**

If you have any questions, queries, suggestions regarding the study please feel free to contact

**Pragya Sharma**

Email:pragyasharma@student.kusms.edu.np, Phone: 9843758178

**Dr. Archana Shrestha**

Email: archana@kusms.edu.np

Phone: 9801002245

For questions about your child's rights as a research participant, or to discuss problems, concerns or suggestions related to the research, or to obtain information or offer input about the research, please contact Institutional Review Committee (IRC), KUSMS.

**Statement of consent**

"I have read this form and I agree for my child's participant in this study."

**Signature of the Participant's Parent:**

Date:

Contact Number of the Participant's Parent:

Signature of Person obtaining Assent:

Date:
