# Supplementary material for: Association of food environment with diet quality and Body Mass Index (BMI) of school-going adolescents in Nepal
Source: PLoS One. 2025 Apr 21;20(4):e0321524. doi: 10.1371/journal.pone.0321524 (PMC12011221; doi:10.1371/journal.pone.0321524)
Supplement: S4 Annex — (WORD) [file pone.0321524.s004.docx]

Namaskar! I am Pragya Sharma, Post Graduate (M.Sc.PH) student in Public Health, Kathmandu University, Dhulikhel. As a course requirement, I am conducting research to understand how the food environment affects diet quality among school-aged adolescents aged 15-19 years. The information collected from you will be useful for this study.

**Please fill in the following blanks:**

Participant's Name: ………………………………………………………………………………

Address: …………………………………………………………………………………………..

Class: …………………………………………………………………………

Name of School: …………………………………………………………………………………

………………………………………………………………………………………………. .....

School Address: ………………………………………………………………………………..

Contact no. (Parents): ……………………………………………………………………..

Provide your answers according to the instructions provided for each set of questions in the following sections.

**Section I: Socio-Demographics**

| Please fill in the blanks or tick the boxes of the correct information about yourself available in the options in the following questions. | | |
| --- | --- | --- |
| S.N. | Questions | Your Answer |
|  | How old are you? | ……………………………………. |
|  | What is your gender? | - Male - Female - Others |
|  | Which ethnicity do you belong to? | - Brahmin/Chhetri - Adibasi/Janajati - Dalit - Terai/Madhesi - Others |
|  | Which religion do you follow? | - Hinduism - Buddhism - Christianity - Muslim - Others |
|  | What is your mother's educational status? | - Illiterate - Literate - Primary level completed (Class 5 pass) - Lower Secondary level completed - ( Class 8 Pass ) - Secondary level completed (SLC/SEE Pass ) - Higher Secondary level completed - ( +2 or equivalent ) - Bachelor's completed - Master's and/or above |
|  | What is your father's educational status? | - Illiterate - Literate - Primary level completed (Class 5 pass) - Lower Secondary level completed - ( Class 8 Pass ) - Secondary level completed (SLC/SEE Pass ) - Higher Secondary level completed - ( +2 or equivalent ) - Bachelor's completed - Master's and/or above |
|  | What is the type of your family? | - Nuclear (parents and their own children only) - Joint (consisting of grandparents, great-grandparents, paternal grandparents, cousins ​​and their children) |
|  | How many members are there in your family? | ………………………………………… |
|  | What is your parent's marital status? | - Parents living together - Father lives away from home - Mother lives away from home - Divorced - One of them has expired |
|  | Which of these media sources do you use the most?  (Select all that apply.) | - Newspaper - Television - Radio - Internet - None |
|  | What is your father's employment status? | - Full-time job - Part-time job - Unemployed |
|  | What is your father's occupation? | - Agriculture - Building Construction - Daily Wages - Government Job - Private job - Business - Home-maker - Foreign Employment |
|  | What is your mother's employment status? | - Full-time job - Part-time job - Unemployed |
|  | What is your mother's occupation? | - Agriculture - Building Construction - Daily Wages - Government Job - Private job - Business - Home-maker - Foreign Employment |
|  | Who earns money in your family? | - Father - Mother - Both - None |

**Section II: Home Food Environment Questionnaire**

You will be asked about different aspects of your home food environment, please read the instructions clearly to fill up the tables and give your answer based upon your understanding and knowledge of the matter.

**Table 1:**

| Places to buy | Mention the time (in minutes) that it usually takes to reach to the nearest … |
| --- | --- |
| Grocery Shop | There is a shop in my own home. By walking: ........ minutes |
| Fruits Vendor | There is a shop in my own home. By walking: ........ minutes |
| Vegetables Vendor | There is a shop in my own home. By walking: …….. minutes |
| Street Food Vendor | There is a shop in my own home. By walking: ……. minutes |

**Table 2: Diet Quality Questionnaire and Home Food Environment**

Please read: Now I’d like to ask you some yes-or-no questions about foods and drinks that you consumed yesterday during the day or night, whether you had it at home or somewhere else. First, I would like you to think about yesterday, from the time you woke up through the night. Think to yourself about the first thing you ate or drank after you woke up in the morning … Think about where you were when you had any food or drink in the middle of the day … Think about where you were when you had any evening meal … and any food or drink you may have had in the evening or late-night... and any other snacks or drinks you may have had between meals throughout the day or night. I am interested in whether you had the food items I will mention even if they were combined with other foods. Please listen to the list of foods and drinks, and if you ate or drank ANY ONE OF THEM, say yes.

| S.N. | Yesterday, did you eat any of the following foods: | Indicate Yes or No. | Yesterday any of the given food was there at your home? |
| --- | --- | --- | --- |
| *1* | Rice, paratha, naan, pau roti, or momo? | - Yes - No | - Yes - No |
| *2* | Roti, whole grain bread, maize, millet, barley, sorghum, buckwheat, or dhindo? | - Yes - No | - Yes - No |
| *3* | Potato, yam, wild yam, or white sweet potato? | - Yes - No | - Yes - No |
| *4* | Daal, chickpeas, beans, soybeans, or quanti? | - Yes - No | - Yes - No |
| *5* | Carrots or ripe yellow pumpkin? | - Yes - No | - Yes - No |
| *6.1* | Saag, spinach, mustard greens, fennel greens, pumpkin shoots, taro leaves, or amaranth greens? | - Yes - No | - Yes - No |
| *6.2* | Gundruk, chamsur palungo, fenugreek greens, or broccoli? | - Yes - No | - Yes - No |
| *7.1* | Tomatoes, cauliflower, cabbage, gourd, or eggplant? | - Yes - No | - Yes - No |
| *7.2* | Bitter gourd, bottle gourd, green pumpkin, lady finger, or radish? | - Yes - No | - Yes - No |
| *8* | Papaya, ripe mango, apricots, or persimmon? | - Yes - No | - Yes - No |
| *9* | Orange, pomelo, grapefruit, or kumquat? | - Yes - No | - Yes - No |
| *10.1* | Apple, banana, avocado, watermelon, mulberries, amla, or guava? | - Yes - No | - Yes - No |
| *10.2* | Grapes, raisins, peaches, plums, pomegranate, Asian pear, or jackfruit? | - Yes - No | - Yes - No |
| *11* | Cakes, biscuits, cookies, donuts, haluwa, jeri or jalebi? | - Yes - No | - Yes - No |
| *12* | Mithai, kheer, chocolates, candies, toffees, or ice cream? | - Yes - No | - Yes - No |
| *13* | Eggs? | - Yes - No | - Yes - No |
| *14* | Paneer or cheese? | - Yes - No | - Yes - No |
| *15* | Dahi, butter milk, or lassi? | - Yes - No | - Yes - No |
| *16* | Sausages, ham, bacon, or canned meat? | - Yes - No | - Yes - No |
| *17* | Goat, mountain goat, lamb or sheep, buffalo, or yak? | - Yes - No | - Yes - No |
| *18* | Local pig or hybrid pig? | - Yes - No | - Yes - No |
| *19* | Chicken, duck, or pigeon? | - Yes - No | - Yes - No |
| *20* | Fish or dried fish? | - Yes - No | - Yes - No |
| *21* | Almonds, peanuts, cashews, pistachios, or walnuts? | - Yes - No | - Yes - No |
| *22* | Chips, Kurekure, Chisbal, Dalmoth, or Bhujiya? | - Yes - No | - Yes - No |
| *23* | Wai Wai? | - Yes - No | - Yes - No |
| *24* | Samosa, pakora, sel roti, puri, or tareko khaja? | - Yes - No | - Yes - No |
| *25* | Milk, tea with milk, or powdered milk? | - Yes - No | - Yes - No |
| *26* | Chiya with sugar, coffee with sugar, milk with sugar, Horlicks or Bournvita? | - Yes - No | - Yes - No |
| *27* | Fruit juice, fruit drinks such as Real or Frooti, or sugar cane juice? | - Yes - No | - Yes - No |
| *28* | Sweet bottled drinks such as Coke, Fanta, or Sprite, or energy drinks such as Red Bull? | - Yes - No | - Yes - No |
| *29* | KFC, Pizza Hut, or other places that serve pizza or burgers? | - Yes - No | - Yes - No |

***Section III: Parental Type and Modeling, Family Meal Practice***

**Table 3:** Please indicate the extent to which you never, sometimes, often, or always do the following.

| **S.N.** | **Statements** | **Never** | **Sometimes** | **Frequently** | **Always** |
| --- | --- | --- | --- | --- | --- |
|  | My parents eat vegetables when I am with them. |  |  |  |  |
|  | My parents eat fruits when I am with them. |  |  |  |  |
|  | My parents eat salad at a restaurant when I am with them. |  |  |  |  |
|  | My parents eat low fat snacks when I am with them. |  |  |  |  |
|  | My parents hear about my problems. |  |  |  |  |
|  | My parents make sure that I tell them where I am going. |  |  |  |  |
|  | My parents usually know where I am after school. |  |  |  |  |
|  | My parents tell me when I do a good job on things. |  |  |  |  |
|  | My parents are interested in my school work. |  |  |  |  |
|  | My parents check to see if I do homework or not. |  |  |  |  |
|  | My parents often ask me what I do with friends. |  |  |  |  |
|  | My parents make me feel better when I am upset. |  |  |  |  |
|  | My parents tell me that they like me just the way I am. |  |  |  |  |
|  | My parents are usually pleased with how I behave. |  |  |  |  |
|  | My parents tell me at what time I must return home. |  |  |  |  |
|  | It is hard for my parents to say ‘no’ to me. |  |  |  |  |
|  | My parents always tell me what to do. |  |  |  |  |
|  | My parents make rules without asking me what I think. |  |  |  |  |
|  | My parents forget the rules that they make for me. |  |  |  |  |
|  | My parents can be talked into things easily. |  |  |  |  |

**Section V: Lifestyle Factors**

**Physical Activity**

Think about all the vigorous activities that you did in the last 7 days. Vigorous physical activities refer to activities that take hard physical effort and make you breathe much harder than normal. Think only about those physical activities that you did for at least 10 minutes at a time.

During the last 7 days, on how many days did you do vigorous physical activities like heavy lifting, digging, aerobics, or fast bicycling?

- _____ days per week
- No vigorous physical activities

If No, Skip to question 42.

How much time did you usually spend doing vigorous physical activities on one of those days?

- _____ hours per day
- _____ minutes per day
- Don’t know/Not sure

Think about all the moderate activities that you did in the last 7 days. Moderate activities refer to activities that take moderate physical effort and make you breathe somewhat harder than normal. Think only about those physical activities that you did for at least 10 minutes at a time.

42. During the last 7 days, on how many days did you do moderate physical activities like carrying light loads, bicycling at a regular pace, or doubles tennis? Do not include walking.

- _____ days per week
- No moderate physical activities
- If No, Skip to question 44.

How much time did you usually spend doing moderate physical activities on one of those days?

- _____ hours per day
- _____ minutes per day
- Don’t know/Not sure

Think about the time you spent walking in the last 7 days. This includes at school and at home, walking to travel from place to place, and any other walking that you have done solely for recreation, sport, exercise, or leisure.

44. During the last 7 days, on how many days did you walk for at least 10 minutes at a time?

- _____ days per week
- No walking

If No, Skip to question 46.

How much time did you usually spend walking on one of those days?

- _____ hours per day
- _____ minutes per day
- Don’t know/Not sure

Think about the time you spent sitting on weekdays during the last 7 days. Include time spent at work, at home, while doing course work and during leisure time. This may include time spent sitting at a desk, visiting friends, reading, or sitting or lying down to watch television.

46. During the last 7 days, how much time did you spend sitting on a week day?

- ____ hours per day
- _____ minutes per day
- Don’t know/Not sure

**Smoking Habit**

Do you currently smoke tobacco on a daily basis, less than daily, or not at all?

- Daily
- Less than daily
- Not at all
- Don’ t want to answer

If ' Not at all' go to question number 49.

On average, how many cigarettes do you currently smoke each week?

……………..per day

……………..per week

49. Have you smoked tobacco daily in the past?

- Yes
- No
- Don’t want to answer
