# Supplementary material for: Association of food environment with diet quality and Body Mass Index (BMI) of school-going adolescents in Nepal
Source: PLoS One. 2025 Apr 21;20(4):e0321524. doi: 10.1371/journal.pone.0321524 (PMC12011221; doi:10.1371/journal.pone.0321524)
Supplement: Attachment — Submitted filename: Response to Reviewers .docx [file pone.0321524.s006.docx]

**Response to Comments:**

1. Please ensure that your manuscript meets PLOS ONE's style requirements, including those for file naming. The PLOS ONE style templates can be found at

<https://journals.plos.org/plosone/s/file?id=wjVg/PLOSOne_formatting_sample_main_body.pdf> and

<https://journals.plos.org/plosone/s/file?id=ba62/PLOSOne_formatting_sample_title_authors_affiliations.pdf>

**Response:** We have checked for templates and inserted line numbers to the document, and we have followed PLOS ONE's style requirements for file naming too.

2. Please amend either the title on the online submission form (via Edit Submission) or the title in the manuscript so that they are identical.

**Response:** The title of the article has been revised as **“Association of food environment with diet quality and Body Mass Index (BMI) of school-going adolescents in Nepal”**, in the manuscript as that in online submission form.

3. Please ensure that you refer to Figure 2 in your text as, if accepted, production will need this reference to link the reader to the figure.

**Response:** The text “**S2** **Figure 2: Body Mass Index (BMI) Chart of the participants**

” is in the Results section (Page number 13).

4. Please remove all personal information, ensure that the data shared are in accordance with participant consent, and re-upload a fully anonymized data set.

**Response:** All the personal information, including name, contact number and also the participant ID have been removed in the re-uploaded data set.

**Response to Reviewers**

**Reviewer 1:**

**Methods**

**Comment:** I did not find Table 1, but your work includes many tables and images, more than the journal allows. So, I recommend including Table 1 in the supporting files.

Which software did you use to do the statistical analysis? You need to mention this in your manuscript.

**Response:** The table is included in supplementary information and named as “**S3 Table: Food groups and the corresponding items”**.

This way we have renumbered tables and we tables numbered 1-4 in the Result section as compared to 1-5 previously.

We have now included the following sentence under the Statistical Analysis section in the Methods to clarify the software used: **"All statistical analyses were conducted using STATA version 14."**

**Results**

**Comment:** I recommend doing more complete title tables, including the place and year of the study.

**Response:** We have revised the titles by adding place and year of study as follows:

**“Table 1: Socio-demographic characteristics of the participants (n = 678) - Budanilkantha, Nepal, 2022**

**Table 2: Characteristics of the Participants' Parents (n=678) - Budanilkantha, Nepal, 2022**

**Table 3: Associations of Home Food Environment with Diet Quality (GDR Score) - Budanilkantha, Nepal, 2022**

**Table 4: Association of Home Food Environment with BMI  - Budanilkantha, Nepal, 2022”**

**Comment:** I did not find Figure 2. And where is Figure 1? You did not mention it in the text?

**Response:** Figure 1 and Figure 2 are included in supplementary information. Figure 1 is named as **S1 Figure 1: Conceptual Framework of the Study** and **S2 Figure 2: Body Mass Index (BMI) Chart of the participants.**

Figure 1 is mentioned in the text in the second last paragraph of Methods section as follows:

**“The conceptual model (S1 Figure 1) illustrates the hypothesized relationships among the home food environment, diet quality, BMI and other covariates that include socio-demographic characteristics and lifestyle factors. It also guided the selection of variables for data collection and informed the data analysis by providing a structured approach to examine the interactions among the assessed variables.”**

Figure 2 is mentioned in the text as **“S2 Figure 2: Body Mass Index (BMI) Chart of the participants” in the Result section.**

**Comment: In Tables 3 and 4, did you think to characterize your sample by GDR score (categorized) and BMI (categorized)? It would be interesting to see if there is some kind of difference.**

**Response:** The GDR score is a score with a range from 0 to 18 that indicates adherence to global dietary recommendations, which include dietary factors protective against non-communicable diseases. [21] We used GDR score in its continuous variable form during the analyses to capture the level of healthy eating and also avoid arbitrary groupings, as this could introduce potential bias. In addition to this, variables in continuous forms allow greater statistical power in our analyses which is why we kept both of the outcome variables (GDR Score and BMI) in their continuous forms in the analyses.

**Comment:** Table 3: In the footnote, include all the aspects you included in model 3 of the home food environment.

**Response:** In table 3, in the footnote, “other aspects of home food environment” has been replaced with “aspects of home food environment  that include home food availability, accessibility to shops, parental modeling and parenting style” and the model 3 has been written as follows:

**“Model 3: Multivariate Analysis of Features of Home Food Environment with Diet Quality (GDR) Score adjusting for Socio-demographics, Physical Activity, Smoking Status and aspects of home food environment  that include home food availability, accessibility to shops, parental modeling and parenting style”**

**Comment:** You also need to make clear the outcome variable of your model. You can put the outcome variable in a line above the models.

Response: In the footnote of table 3, this text has been added: **“Outcome variable: Global Dietary Recommendations (GDR) score indicating the diet quality”**

In the footnote of table 4, this text has been added: **“Outcome variable: Body Mass Index (BMI)”**

**Comment:** Based on my comments on Table 3, I recommend revising Table 4. You do not put a footnote in Table 4.

**Response:** Footnotes have been added to table 4 as well just like that in table 3 as follows:

**“* Model 1: Bivariate Analysis of Features of Home Food Environment with Diet Quality (GDR) Score**

**** Model 2: Multivariate Analysis of Features of Home Food Environment with Diet Quality (GDR) Score Adjusting for Socio-demographics, Physical Activity and Smoking Status**

***** Model 3: Multivariate Analysis of Features of Home Food Environment with Diet Quality (GDR) Score adjusting for Socio-demographics, Physical Activity, Smoking Status and aspects of home food environment  that include home food availability, accessibility to shops, parental modeling and parenting style”**

**Discussion**

**Comment:** I miss citations of studies about the importance of the school environment in adolescents’ food consumption. In the third paragraph, you did not cite one article to discuss this theme; in the next paragraph, when you cited this theme again, you did not mention any previous work.

**Response:** Thank you for notifying us of this. In the third paragraph of the discussion section, the citation has been added to this text:

“It did not consider the distance from their schools, which could also play a significant role in their eating habits, as adolescents might spend a significant portion of their day at school **[29]** and could purchase food from vendors near their school instead of near their home as the accessibility of street food in neighborhoods close to schools may have a big impact on their dietary choices. **[32]”**

In the fourth paragraph of the discussion section, the citation has been added to this text:

“These contradicting results may be influenced by other factors, such as the consumption of food outside the home, particularly at school, which can affect diet quality regardless of what is available at home**. [32]”**

**Comment:** In your discussion, you cite other works that found similar results. However, I miss you discussing possible reasons for the findings you found, reasons that could justify why you see some patterns in your sample.

**Response:** Thank you for the comments. We have addressed it by adding additional texts explaining the reasons for the patterns in our sample. The new texts are pasted below and presented in bold text:

“Adolescents had lower diet quality scores when they lived at a walking distance of less than 10 minutes or greater than 20 minutes to a vegetable shop; this association did not exist for accessibility of fruit shops. Studies [26], [27] have documented that increased accessibility was positively associated with fruit and vegetable intake in children, but this was not seen in our findings. **A number of contextual factors may explain this discrepancy.** One possible explanation for the lower diet quality associated with vegetable shops farther away is that these places may have fewer overall healthy food alternatives or be located in communities with limited access to fresh produce markets. **Furthermore, greater distances to vegetable shops could exacerbate the challenges associated with transportation, time constraints, and economic barriers to purchasing fresh produce.**  This could lead to a general lack of access to a diverse range of fresh and healthy foods, not only vegetables, resulting in a lower quality diet.

Adolescents also had lower diet quality scores when they lived within 10 minutes or > 20 minutes from street food vendors. However, it's important to note that the study only measured the accessibility to street food shops from the adolescents' homes. It did not consider the distance from their schools, which could also play a significant role in their eating habits, as adolescents might spend a significant portion of their day at school **[29]** and could purchase food from vendors near their school instead of near their home **as the accessibility of street food in neighborhoods close to schools may have a big impact on their dietary choices. [32]**

**One of the intriguing findings in our study was the negative association between availability of starchy foods, particularly rice, and BMI.** Starchy foods, particularly rice, are part of the staple diet in almost every Nepalese household.  Everyday availability of certain foods at home is expected to affect the diet pattern and hence the BMI, as seen in a national US study [35]. Starchy food items such as rice contain carbohydrates which are known to contribute to increasing the body weight and would raise the BMI [36] but we found that home availability of a starchy staple in the past day was associated with a lower BMI. **This unexpected finding may be attributed to the small number of adolescents (15 out of 678) who reported not having starchy staples available at home, which limits the generalizability of the result. Other dietary factors such as portion sizes and eating frequency that this study did not measure may explain this paradoxical finding.  Hence, this finding should be interpreted with caution, as the small sample of respondents without availability of starchy staples at home may not adequately represent broader trends within the population.**

 Positive parental modeling was associated with a higher BMI. Since the majority of our study participants were either underweight (41%) or had a normal weight (47%), a higher BMI might indicate better health status in our study population. **Adolescent Nutrition Survey in Nepal, 2014 indicated that 42.2 % of adolescents aged 15-19 years were underweight, 55.9 % had a normal weight and only 1.8 % were overweight. [18] The NDHS 2022 also reported that only 5% of the adolescent girls aged 15-19 years were overweight and less than 1% were obese. [24] These data suggest that higher BMI likely indicates better health status in our population, hence, this finding is in expected direction as better eating practices of parents is suggested by positive parental modeling.”**

**Comment:** You only mention the conceptual framework in the discussion. It should be mentioned in your methods and explain how you use it. Furthermore, citing the supplementary material in the text is recommended so the readers can go to the site and learn that these files exist and learn more about your work.

**Response:** Apologies for the confusion. The conceptual framework mentioned in the discussion section is not that of the study but of the food environment. We clarified this further in the second last paragraph of our discussion section and have also provided citation to the supplementary material as follows:

“There are several strengths to this study. First, to our knowledge, the food environment has not been studied before in Nepalese adolescents and this study is first of its kind in Nepal. Second, for our characterization of the food environment, assessment of home food availability and diet quality, and measurement of parenting style, we have used validated conceptual frameworks of the food environment **[10]** and tools along with expert suggestions, and pretested all tools that were used.” and citation of the conceptual framework has been added too.

Also, details on conceptual framework has been added in the methods section in the second last paragraph as follows:

**“A conceptual framework was developed to guide the study, illustrating the relationship between home food environment, diet quality, and BMI among adolescents. The framework allowed identification of the variables and their interaction among each other.**

**The conceptual model (Figure 1) illustrates the hypothesized relationships among the home food environment, diet quality, BMI and other covariates that include socio-demographic characteristics and lifestyle factors. It also guided the selection of variables for data collection and informed the data analysis by providing a structured approach to examine the interactions among the assessed variables.”**

**Reviewer 2:**

**Abstract:**

**Comment:** Well-written and consistent. I believe the **conclusion could be phrased more effectively.**

**Response:** Thank you. We have rephrased the conclusion in abstract as follows:

**“This study highlights the impact of home food environment on diet quality and BMI among adolescents in urban Nepal. Availability of healthy foods positively affects diet quality, while that of unhealthy items negatively influences it. Future research should explore broader food environmental factors and intervention strategies to improve adolescents’ diet quality.”**

**Introduction:**

**Comment:** Clearly presents and connects the facts related to the topic. However, I felt the need for a deeper exploration of the concepts related to the home food environment, as addressed in Gálvez-Espinoza’s (2017) conceptual model, which explores the food environment in Low- and Middle-Income Countries (LMICs).

**Response:** Thank you for the comment. Regarding concepts on food environment, we have accessed info from the paper titled “Concepts and critical perspectives for food environment research: A global framework with implications for action in low- and middle-income countries” and its reference has been provided in our paper. Turner C, Aggarwal A, Walls H, Herforth A, Drewnowski A, Coates J, et al. Concepts and critical perspectives for food environment research: A global framework with implications for action in low- and middle-income countries. Global Food Security. 2018;18: 93–101. doi:10.1016/j.gfs.2018.08.003

We have added the following text in the third paragraph of introduction section to clarify this more:

**“The food environment influences food acquisition and consumption of the individuals. It includes external factors such as food availability and price as well as individual determinants like accessibility and affordability.[10]”**

**Comment:** Additionally, it lacks an overview of the nutritional status of adolescents in Nepal, which would better contextualize the findings in the discussion.

**Response:** Thank you for the comment. We have added the following information on this in the third last paragraph of the introduction section:

Regarding overview of the nutritional status of adolescents in Nepal, this info has been added in the third last paragraph of Introduction section:

**“The Nepal Demographic and Health Survey 2022 reports that among adolescent girls aged 15-19 years, 27% are of short stature (indicating stunting), 26% are thin (indicating undernutrition), 5% are overweight, and less than 1% are obese, while among adolescent boys of the same age group, 7% are of short stature (indicating stunting), 41% are thin (indicating undernutrition), 4% are overweight, and 3% are obese. [25] Adolescent Nutrition Survey in Nepal, 2014 indicated that 42.2 % of adolescents aged 15-19 years were underweight, 55.9 % had a normal weight and only 1.8 % were overweight. [18]”**

**Comment:** Moreover, the second paragraph is somewhat confusing. I suggest using this paragraph to discuss the GDR and its relevance to this type of study or why assessing diet quality is important in this population.

**Response:** This paragraph has been added as the second last paragraph in the Introduction section:

“**The double burden of malnutrition is rising among Nepalese adolescents [25]. GDR score is an easy to calculate indicator for the assessment of their diet quality. The score indicates adherence to global dietary recommendations, which include dietary factors protective against non-communicable diseases. The higher the GDR score, the more recommendations are likely to be met. [22] Understanding diet quality in this population is essential, as it informs targeted interventions aimed at improving nutrition and addressing the growing concerns of both underweight and overweight adolescents in Nepal.”**

**Comment:** Nonetheless, after reading the introduction in its entirety, the motivations for conducting the study are clear.

**Response:** Thank you.

**Methods:**

**Comment:** Comprehensive and explanatory, allowing for easy replication of the study. My suggestion is to clarify exactly which sociodemographic variables were used in each regression model, as there are many.

**Response:** In the methods section, in the second last paragraph titled “Other Covariates”, sociodemographic variables are named with the text:

**“We assessed the socio-demographic characteristics of the participants (age, gender, ethnicity, religion, family type, and size) and participants’ parents (Marital status, Employment Status, Occupation, Education and Family Income status) using Nepal Demographic Health Survey questionnaires.”**

And this sentence has been added in the same paragraph that clarifies sociodemographic variables used in each regression model:

**“All these socio-demographic variables have been included for adjustment in model 2 and 3 of data analyses.”**

**Results:**

**Comment:** The results are clearly presented and support the subsequent discussions. Regarding the tables, the captions could be more explicit, in line with the earlier suggestion about detailing the sociodemographic variables used in each regression model.

**Response:** The adjusted sociodemographic variables are clarified in the methods section as explained above.

We have added the following text for more clarity in the footnotes of Tables 3 and 4.

* Model 1: Bivariate Analysis of Features of Home Food Environment with Diet Quality (GDR) Score

** Model 2: Multivariate Analysis of Features of Home Food Environment with Diet Quality (GDR) Score adjusting for Socio-demographics, Physical Activity and Smoking Status

*** Model 3: Multivariate Analysis of Features of Home Food Environment with Diet Quality (GDR) Score adjusting for Socio-demographics, Physical Activity, Smoking Status and **aspects of home food environment that include home food availability, accessibility to shops, parental modeling and parenting style**

**Comment:** Additionally, what is the relevance of the data on the "most popular media source"? It is shown in Table 1 but was not used in any analysis. Was there a hypothesis regarding this variable?

**Response:** Thank you for the comment. We collected this information as a descriptive variable to describe the context. We have not tested any hypothesis related to this, nor was it included in any regression analysis.

**Discussion:**

**Comment:** The discussion addresses all the results and corroborates the findings with prior literature, but I found the text a bit tedious due to its repetitive structure in each paragraph: presenting the finding in one sentence, bringing in one or two studies that support or contradict it, and then offering hypotheses. I suggest rewriting and better connecting the paragraphs for smoother reading.

**Response:** We have revised the discussion section and the changes that have been made are presented in the bold text as follows:

“In an urban area in Nepal, **the diet quality of adolescents is likely to be influenced by accessibility to different types of shops.** **Our findings indicate that** those who have to walk longer distances to get to vegetable shops and street food vendors tend to have lower-quality diets. Additionally, having salty or fried snacks and processed meat available at home is associated with poorer diet quality among adolescents. Conversely, when fruits and vegetables are available at home, teenagers will likely have a better diet. Parenting modeling and parenting style are not related to adolescent’s diet quality. Being close to vegetable shops and having starchy staples available at home is linked to lower body mass index (BMI). Positive parental modeling, meaning when parents demonstrate healthy eating behaviors, is associated with teenagers having higher BMI.

Adolescents had lower diet quality scores when they lived at a walking distance of less than 10 minutes or greater than 20 minutes to a vegetable shop; this association did not exist for accessibility of fruit shops. Studies [27], [28] have documented that increased accessibility was positively associated with fruit and vegetable intake in children, but this was not seen in our findings. **A number of contextual factors may explain this discrepancy.** One possible explanation for the lower diet quality associated with vegetable shops farther away is that these places may have fewer overall healthy food alternatives or be located in communities with limited access to fresh produce markets.**Furthermore, greater distances to vegetable shops could exacerbate the challenges associated with transportation, time constraints, and economic barriers to purchasing fresh produce.** This could lead to a general lack of access to a diverse range of fresh and healthy foods, not only vegetables, resulting in a lower quality diet.

Adolescents also had lower diet quality scores when they lived within 10 minutes or > 20 minutes from street food vendors. However, it's important to note that the study only measured the accessibility to street food shops from the adolescents' homes. It did not consider the distance from their schools, which could also play a significant role in their eating habits, as adolescents might spend a significant portion of their day at school **[29]** and could purchase food from vendors near their school instead of near their home **as the accessibility of street food in neighborhoods close to schools may have a big impact on their dietary choices. [32]**

In our study, adolescents who had home availability of salty or fried snacks and processed meat in the past day had lower diet quality. In comparison, those with the availability of fruits and vegetables had higher diet quality. Previous studies [30], [31] have found similar associations between availability and adolescent consumption of various food items. The availability of fruits and vegetables at home is associated with a higher likelihood of their consumption, leading to improved diet quality. Conversely, the presence of processed foods at home tends to deteriorate diet quality. [5] However, our study found no significant association between the availability of other healthy and unhealthy food items and diet quality. These contradicting results may be influenced by other factors, such as the consumption of food outside the home, particularly at school, which can affect diet quality regardless of what is available at home. **[32]**

There was no significant association between parental modeling and parenting style and diet quality.  **This finding is consistent** with a study conducted in adolescent-parent pairs [13] reported that parental modeling and parenting style were not directly associated with adolescent dietary intake, but they may indirectly shape what foods are made available at home. In contrast to previous studies [33], we did not identify any associations of diet quality with the parenting style. Our findings may differ because our study focused on 15-19-year-olds, who are in late adolescence and have greater autonomy in their choices compared to younger adolescents aged 10-14 years.**[34]**

**One of the intriguing findings in our study was the negative association between availability of starchy foods, particularly rice, and BMI.** Starchy foods, particularly rice, are part of the staple diet in almost every Nepalese household.  Everyday availability of certain foods at home is expected to affect the diet pattern and hence the BMI, as seen in a national US study [35]. Starchy food items such as rice contain carbohydrates which are known to contribute to increasing the body weight and would raise the BMI [36] but we found that home availability of a starchy staple in the past day was associated with a lower BMI. **This unexpected finding may be attributed to the small number of adolescents (15 out of 678) who reported not having starchy staples available at home, which limits the generalizability of the result. Other dietary factors such as portion sizes and eating frequency that this study did not measure may explain this paradoxical finding.  Hence, this finding should be interpreted with caution, as the small sample of respondents without availability of starchy staples at home may not adequately represent broader trends within the population.**

Positive parental modeling was associated with a higher BMI. Since the majority of our study participants were either underweight (41%) or had a normal weight (47%), a higher BMI might indicate better health status in our study population. **Adolescent Nutrition Survey in Nepal, 2014 indicated that 42.2 % of adolescents aged 15-19 years were underweight, 55.9 % had a normal weight and only 1.8 % were overweight. [18]**

**The NDHS 2022 also reported that only 5% of the adolescent girls aged 15-19 years were overweight and less than 1% were obese. [25] These data suggest that higher BMI likely indicates better health status in our population, hence, this finding is in expected direction as better eating practices of parents is suggested by positive parental modeling.”**

**Comment:** I am uncertain about the conclusion that positive parental modeling is associated with higher BMI being a healthy pattern. What is the predominant nutritional status of adolescents in the same age group in the country? Is there any population-based study that supports this conclusion? I did not find references cited in this paragraph.

**Response:** The seventh paragraph of the discussion section has been revised, the revised portion is made bold here:

“Positive parental modeling was associated with a higher BMI. Since the majority of our study participants were either underweight (41%) or had a normal weight (47%), a higher BMI might indicate better health status in our study population. **Adolescent Nutrition Survey in Nepal, 2014 indicated that 42.2 % of adolescents aged 15-19 years were underweight, 55.9 % had a normal weight and only 1.8 % were overweight. [18]**

**The NDHS 2022 also reported that only 5% of the adolescent girls aged 15-19 years were overweight and less than 1% were obese. [25] These data suggest that higher BMI likely indicates better health status in our population, hence, this finding is in expected direction as better eating practices of parents is suggested by positive parental modeling.”**

**Conclusion:**

**Comment:** Clear and address the study question while proposing future studies to address questions this study could not answer.

**Response:** Thank you. These sentences have been added in the conclusion:

**“This study was limited to the home food environment and hence doesn’t capture the wider areas of food environments that adolescents encounter. Given that they are also influenced by factors such as the school food environment, social media, and peer interactions, among others, future studies should consider these dimensions to gain a more comprehensive understanding of their dietary behaviors.”**

**Comment:** Regarding the writing, ensure that all parentheses are properly opened and closed.

**Response:** We have rechecked all the parentheses and made sure that they are properly opened and closed.
